# CD163^+^ Foamy Macrophages Are Associated with the Morphogenesis of Oral Verruciform Xanthoma through Angiogenesis by VEGF Expression: An Immunohistochemical Study

**DOI:** 10.3390/dj8010018

**Published:** 2020-02-14

**Authors:** Manabu Shigeoka, Yu-ichiro Koma, Takayuki Kodama, Mari Nishio, Masaya Akashi, Hiroshi Yokozaki

**Affiliations:** 1 Division of Pathology, Department of Pathology, Kobe University Graduate School of Medicine, 7-5-1 Kusunoki-cho, Chuo-ku, Kobe 650-0017, Japan; 2 Division of Oral and Maxillofacial Surgery, Department of Surgery Related, Kobe University Graduate School of Medicine, 7-5-2 Kusunoki-cho, Chuo-ku, Kobe 650-0017, Japan

**Keywords:** oral verruciform xanthoma, foamy macrophage, CD163, angiogenesis, VEGF

## Abstract

Oral verruciform xanthoma (OVX) is an uncommon benign lesion that is characterized histologically by the accumulation of several foamy macrophages in the lamina propria papillae. The pathogenesis of OVX has not been completely elucidated, although the significance of macrophage polarization (M1, tumor suppression; and M2, tumor promotion) and the contribution of M2 macrophages to angiogenesis are well established. This study investigated the role of foamy macrophages in OVX, with a focus on angiogenesis. Four patients who underwent surgical excision or total excisional biopsy for OVXs were enrolled in this study. We evaluated the expression of the macrophage markers CD68 (broad) and CD163 (M2) and the CD34-positive microvessel density (MVD) of OVXs. The foamy macrophages of all patients exhibited positivity to CD68 and CD163. We evaluated the MVD and the expression of the vascular endothelial growth factor (VEGF) based on histological architecture. The MVD of all OVX cases was significantly higher than that of the corresponding normal epithelia. Interestingly, the MVD of verrucous-type OVX cases was higher than that of the other type. VEGF was expressed on foamy macrophages in all cases. Overall, the foamy macrophages expressing CD163 were associated with the morphogenesis of OVX through the process of angiogenesis by VEGF expression.

## 1. Introduction

Oral verruciform xanthoma (OVX) is a rare benign lesion that was first reported in 1971 [1]. Microscopically, OVX is characterized by papillary or verrucous proliferation of squamous epithelium with keratosis and several foamy macrophages accumulated in the lamina propria papillae.

The pathogenesis of OVX remains unclear. Several researchers have reported that OVX is a reactive lesion with lipid-containing macrophages resulting from epithelial cell degeneration [2,3,4,5]. On the other hand, it has been suggested that the verrucous and papillary epithelial architecture of OVX is secondary to the presence of foamy macrophages, which affects the metabolism of the epithelial cells, thereby leading to keratotic changes [6].

From an oncogenic viewpoint, macrophages are divided into M1 (tumor suppression) and M2 (tumor promotion) [7,8,9]. M2 macrophages exhibit specific receptors such as hemoglobin scavenger receptors (CD163), macrophage scavenger receptor I (CD204), and mannose receptors (CD206) [10]. Several researchers have demonstrated that M2-macrophage-derived humoral factors induce angiogenesis [11,12]. We have recently reported about the significance of CD163^+^ macrophages in oral carcinogenesis [13,14]. However, to the best of our knowledge, there are no reports investigating the pathogenesis of OVX focusing on the role(s) of macrophages in angiogenesis in particular.

On the basis of the abovementioned background, we tested our hypothesis that foamy macrophages are linked to the morphogenesis of OVX via angiogenesis.

## 2. Materials and Methods

### 2.1. Clinical Information

Five OVX cases were extracted from the electronic database of the Kobe University Hospital Pathology System using the keyword “verruciform xanthoma”. We enrolled four patients who underwent surgical excision or excisional biopsy at the Department of Oral and Maxillofacial Surgery, Kobe University Hospital (Kobe, Japan), from 2001 to 2015. Patients with OVX who were diagnosed only by local biopsy were excluded. All subjects provided the written informed consent to release clinical information and samples for retrospective study. Furthermore, this study was approved by the Kobe University Institutional Review Board (approval number: B190043 on 29 May 2019).

The clinical data of the four patients with OVX were retrospectively investigated for sex, age, region, clinical diagnosis of the lesion, and previous medical history using their clinical records.

### 2.2. Morphological Evaluation

All resected specimens were fixed in 10% formalin and embedded in paraffin. All cases were classified into three types (verrucous, papillary, and flat types) as described previously [6,15]. The histological diagnosis and classification of all patients was confirmed after reviewing the hematoxylin and eosin (HE)-stained slides by three pathologists (M.S., Y.-i.K., and M.N.).

### 2.3. Immunohistochemical Evaluation

Immunohistochemistry was performed using the EnVision Dual Link System-HRP,3,3-diaminobenzidine (DakoCytomation, Glostrup, Denmark). Specific mouse monoclonal antibodies to CD68 (1:100, #Kp-1, Dako), CD163 (1:100, #10D6, Novocastra, Newcastle upon Tyne, UK), CD34 (1:50, #NU-4A1, Nichirei, Tokyo, Japan), and rabbit polyclonal antibody to vascular endothelial growth factor (VEGF) (1:100, #A-20, Santa Cruz Biotechnology, Santa Cruz, CA, USA) were used for the primary reaction. All immunohistochemical evaluations were performed by three pathologists (M.S., Y.-i.K., and T.K.) with their clinical information blinded.

### 2.4. Assessment of Microvessel Density

The microvessel density (MVD) was evaluated using the anti-CD34 antibody within 100 μm of the stroma from the superficial epithelium. The sections were screened on a 200× field, and three areas with the highest number of positively stained capillaries or venules with lumens were selected. Vessels with obscured outlines at the margins were excluded. MVD was evaluated by calculating the average numbers of the three fields according to a previous report [16].

### 2.5. Statistical Analysis

Statistical significance was analyzed using the SPSS Statistics version 22 (IBM, Chicago, IL, USA). The comparisons of MVDs were analyzed using the paired or student’s *t*-tests. In this study, *p* values < 0.05 were considered statistically significant.

## 3. Results

### 3.1. Clinical Findings

Table 1 shows the clinical information of patients with OVX enrolled in this study. Of the four patients, three were men and one was a woman. The age range of the patients at the time of histopathological examination was 15–54 years (mean 40.0 years). Three patients had OVX in the gingiva and one patient had it in the hard palate. The size of the OVXs ranged from 3.0 to 5.0 mm (mean 4.3 mm). The suggested clinical diagnoses were epulis (Case 1), gingival tumor (Case 2), squamous papilloma (Case 3), and erosion (Case 4). Three patients had previous medical histories. Case 1 had been diagnosed with pneumonia and macroscopic hematuria 9 years before his first visit to our hospital. Case 2 had dyslipidemia and hyperuricemia, which were treated with medications. Case 3 had no previous medical history. Finally, Case 4 had appendicitis and Basedow’s disease.

### 3.2. Morphological Findings

**Figure 1:**
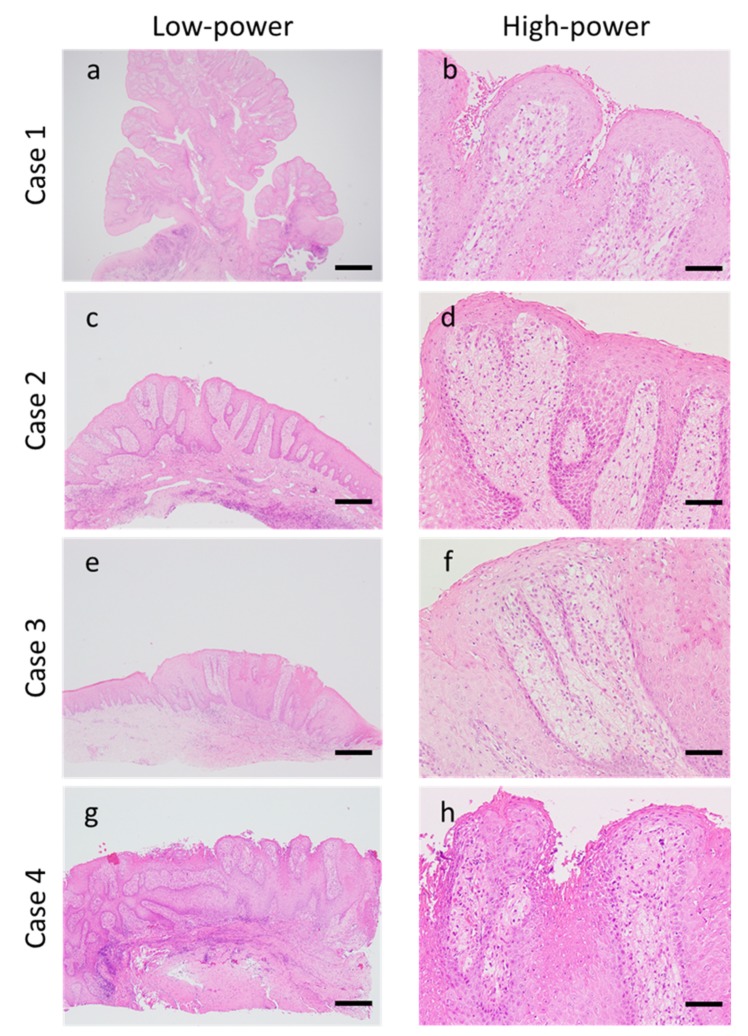
Morphological findings of oral verruciform xanthoma. Epithelial acanthosis with no atypia was observed in all patients with oral verruciform xanthomas (OVXs). (**a**,**c**,**e**,**g**) Chronic inflammatory cell infiltration into the connective tissue was observed. (**b**,**d**,**f**,**h**) Several foamy cells were accumulated in the connective tissue papillae. Case 1 exhibited verrucous-type OVX ((**a**): scale bar, 500 µm; original magnification, 20×; (**b**): scale bar, 50 µm; original magnification, 200×). Case 2–4 exhibited flat-type OVX ((**c**,**e**,**g**): scale bar; 200 µm, original magnification: 40×, (**d**,**f**,**h**): scale bar; 50 µm, original magnification; 200×).

Morphological evaluation was performed using HE-stained specimens. Epithelial acanthosis with no atypia was observed in all patients with OVXs. In all patients, numerous foamy cells were accumulated in the connective tissue papillae, and mild-to-moderate degree of chronic inflammatory cells infiltration containing primarily lymphocytes into the connective tissue was observed. Case 1 alone was classified into the verrucous type, which is characterized by an elevated and well-circumscribed architecture. The remaining three cases were classified as flat type, which exhibits a flat surface and epithelial proliferation below the surface (Figure 1a–h and Table 1).

### 3.3. Expression of Macrophage Markers in OVX

**Figure 2:**
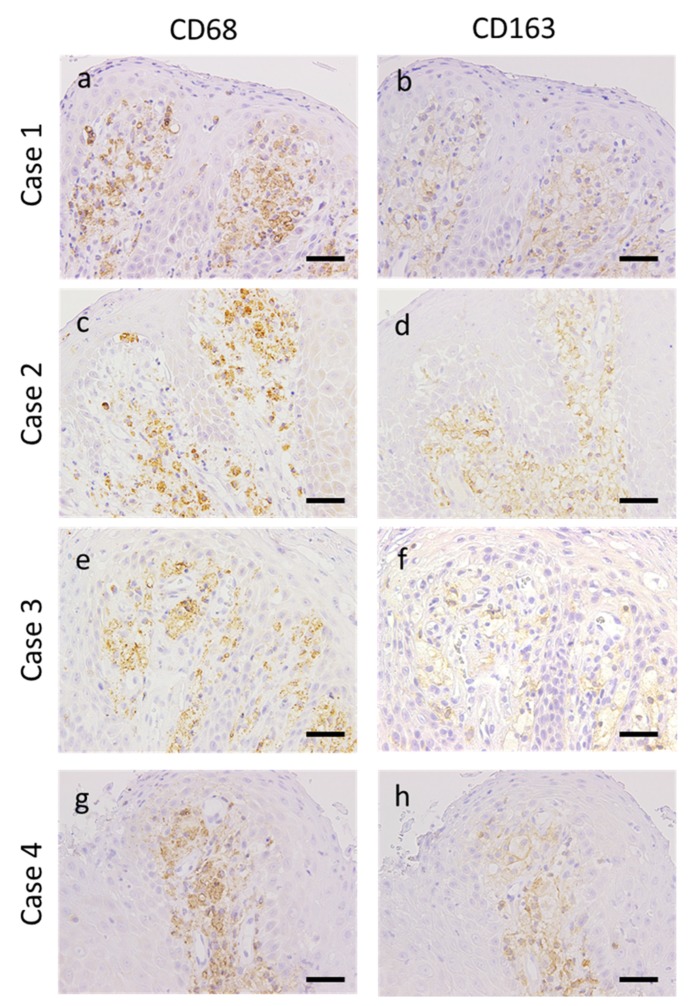
Immunoreaction of macrophage markers in patients with oral verruciform xanthoma. Representative images of CD68 and CD163 expression in oral verruciform xanthoma. (**a**,**c**,**e**,**g**) CD68 was positive in the foamy cells of all patients with a cytoplasmic pattern. (**b**,**d**,**f**,**h**) CD163 was positive in the foamy cells of all patients with a membranous pattern (scale bar, 20 µm; original magnification, 400×).

The expressions of CD68 (broad macrophage marker) and CD163 (M2 macrophage maker) were positive with a cytoplasmic and membranous pattern respectively in the foamy cells of all patients with OVXs. Furthermore, there was no significant difference in the expression of these markers between the verrucous and flat-type OVXs (Figure 2a–h).

### 3.4. Angiogenesis in OVX

**Figure 3:**
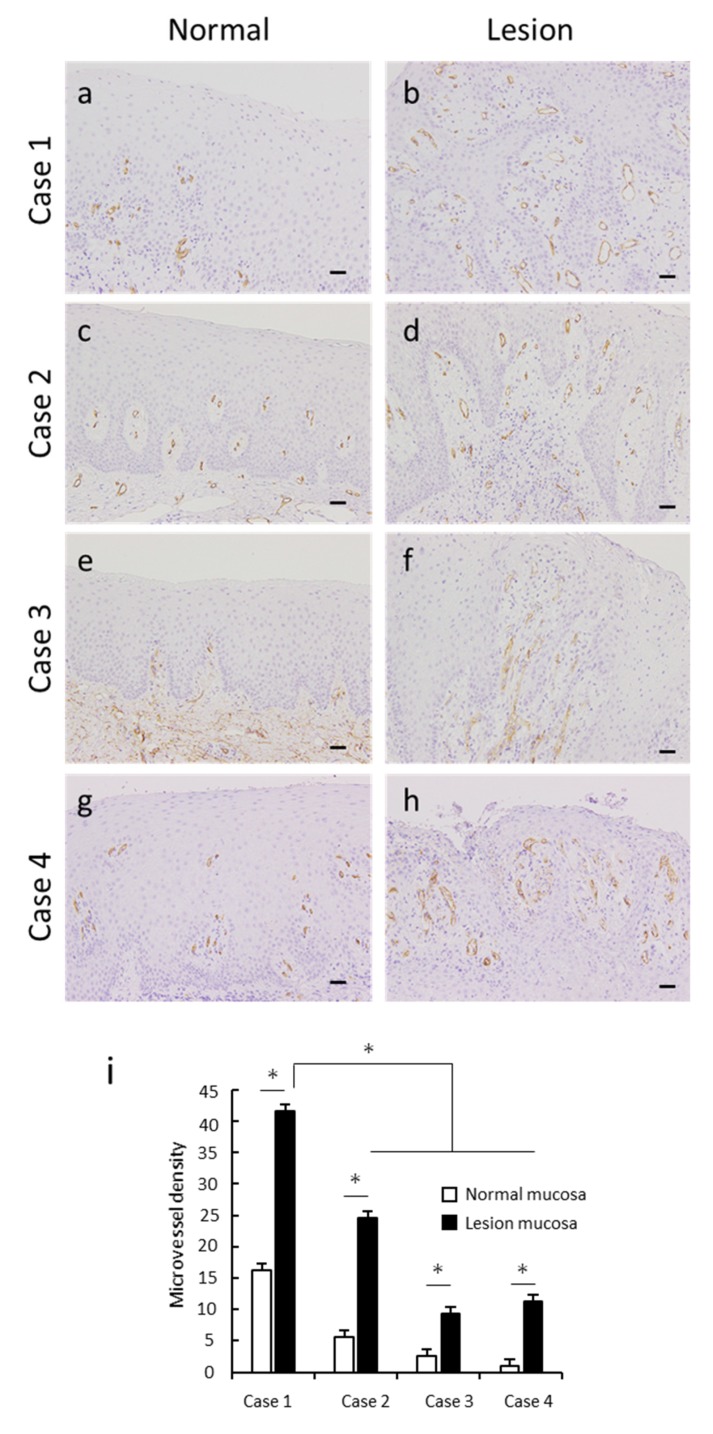
Angiogenesis in oral verruciform xanthoma. Representative CD34 images of oral verruciform xanthoma. (**a**,**c**,**e**,**g**) Few CD34-postive capillaries and small vessels with lumens were observed in the normal mucosa of all patients. (**b**,**d**,**f**,**h**) Vascularization of several elongated small blood vessels was observed in the connective tissue papillae in the lesioned mucosa of all patients (scale bar, 50 µm; original magnification, 200×). (**i**) Microvessel density (MVD) of all oral verruciform xanthoma cases was significantly higher than that of the corresponding normal epithelia. MVD was evaluated within 100 μm of the stroma from the superficial epithelium. The MVD of verrucous-type OVX was higher than that of the flat-type cases. CD34-postive capillaries and small vessels with lumens were selected, and the numbers of microvessels were counted as MVD (per 200× field). * *p* < 0.05 by *t*-test.

M2 macrophages exert important functions in tumor angiogenesis [17,18]. Therefore, we confirmed the process of angiogenesis in OVXs using the anti-CD34 antibody. Vascularization and elongation of several small vessels were not noticeable in normal epithelia but were visible in OVX epithelia (Figure 3a–h). Statistically, the MVDs of OVXs in all patients were significantly higher than those of the corresponding normal epithelia, and the MVD of verrucous-type OVX (Case 1) was higher than that of the flat-type cases (Figure 3i).

### 3.5. Expression of VEGF in the OVX Microenvironment

**Figure 4:**
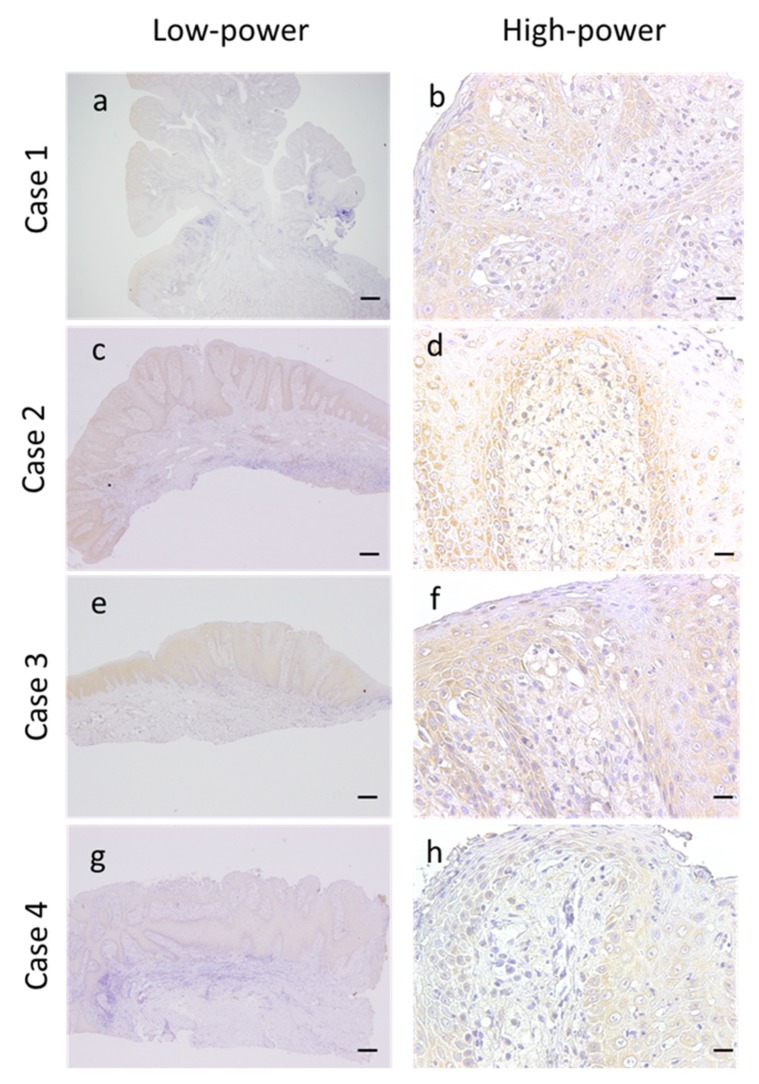
Vascular endothelial growth factor (VEGF) immunostaining in oral verruciform xanthoma. Representative VEGF images of oral verruciform xanthomas. (**a**,**c**,**e**,**g**) Low-power view of VEGF images. The expression level of VEGF in the epithelium of OVX was equal to that of the corresponding normal epithelium in all patients (scale bar, 1000 µm; original magnification; 12.5×). (**b**,**d**,**f**,**h**) High-power view of VEGF images. VEGF also exhibited positive staining in the foamy macrophages in all patients. However, in Case 4, the immunoreactivity was weaker than that in other cases (scale bar, 20 µm; original magnification, 400×).

We subsequently examined the expression of angiogenic inducer in the OVX microenvironment using the anti-VEGF antibody. Results of immunohistochemistry demonstrated that not only epithelial cells but also foamy cells expressed VEGF in the OVX microenvironment. Furthermore, no differences were observed in the expression levels in the epithelia of normal and lesioned regions (Figure 4a–h). Foamy cells of the flat-type OVX (Case 4) demonstrated weak immunoreactivity compared with that of the other cases (Figure 4h).

## 4. Discussion

According to previous reports, the incidence rate of OVX within a 12-year-period was 0.025%–0.094% [19,20]. In a study by Tamiolakis et al. (2018), 13 patients with OVX represented approximately 0.04% of 35,617 samples accessioned from 1971 to 2017 [21]. In this study, approximately 0.06% of 6499 cases were histologically confirmed as being OVX. Therefore, our results are consistent with those of previous reports and thus suggest that OVX is an uncommon lesion.

Previous reports [6,15,22] have demonstrated that OVXs developed in the masticatory mucosa, such as the gingiva and hard palate, with stromal inflammatory changes, as observed in the present study. Consistent with previous reports [23], we observed higher reactivity for CD3 compared with CD20 in the lymphocytes that had infiltrated into all OVXs analyzed here (data not shown). Ide et al. (2008) suggested periodontal pathogens, mechanical stimuli, smoking, alcohol, drugs, and sensitizing or allergic agents of foodstuffs and dental materials are involved in OVX [23]. Belknap et al. (2020) noted subepithelial inflammation, which supports the hypothesis that inflammation is the main etiology of OVX [24]. Moreover, de Andrade et al. (2015) concluded that inflammation plays a role in the development of OVX [15]. Furthermore, accumulation of foamy macrophages was observed in the superficial, rather than the deep, area of the lamina propria. This finding is consistent with that of a previous report [1]. We also confirmed that abnormal Ki-67 expression was not observed in the squamous epithelium and foamy cells of our four cases (data not shown). Based on the reasons stated above, it is reasonable to speculate that inflammation caused by mechanical stimulation plays a vital role in the development of OVX.

Conversely, a few researchers have reported an association between OVX and systemic diseases, including hyperlipidemia, graft-versus-host disease, and congenital hemidysplasia with ichthyosiform nevus and limb defects syndrome [25,26,27]. In our study, only Case 2 had dyslipidemia. We thus may not discount the association of OVX with systemic diseases.

Macrophages are one of the major inflammatory cells. CD163, a hemoglobin scavenger receptor, is expressed on monocytes and macrophages [28]. In the present study, the majority of foamy macrophages of OVXs expressed not only the broad macrophage marker CD68 but also the M2 macrophage marker CD163. Consistent with our findings, de Andrade also reported that the foamy macrophages of OVX expressed the marker CD163 [15]. However, the role of CD163^+^ foamy macrophages of OVX has not yet been completely elucidated.

In the tumor microenvironment, M2 macrophages play critical roles in the process of angiogenesis through the secretion of VEGFA, epidermal growth factor, and interleukin-8 [12,17,29,30]. We have also previously demonstrated that M2 macrophages contribute to the process of angiogenesis in the esophageal squamous cell carcinoma microenvironment [18]. In addition, several researchers have reported that CD163^+^ macrophages promote angiogenesis in oral squamous cell carcinoma [31,32,33]. On the other hand, to our knowledge, there is a lack of reports on angiogenesis in OVX.

In this study, the MVDs of OVX were found to be significantly higher than those of the corresponding normal mucosa. We evaluated the expression of VEGF in OVX for the first time and found that both epithelial cells and foamy macrophages expressed VEGF. There were no differences in the expression levels of VEGF in the epithelia of normal and lesioned mucosa. Thus, the expression of VEGF in the epithelium may not be related to OVX morphogenesis. We speculate that VEGF expression from foamy macrophages plays an important role in angiogenesis in OVX. In fact, we observed a rich density of CD34-positive microvessels within the area of foamy macrophage accumulation. Interestingly, compared with the other types, verrucous-type OVX had a significantly higher MVD. The difference in MVD of OVX might be associated with the differences in the histological architecture. Conversely, we could not provide an explanation for the lower expression level of VEGF observed in Case 4 compared with the remaining cases. The limitations of our study include its very limited sample size (*n* = 4) and the absence of papillary-type OVX. To overcome these drawbacks, it may be necessary to conduct additional studies using a larger sample size.

Our inability to clarify the mechanism of angiogenesis in OVX was another limitation of our study. Previously, Nakayama et al. (2015) suggested that polypoid colorectal adenomas have conglomerates of vessels, whereas the structure and distribution of submucosal vessels were identical to those of normal mucosa in flat colorectal adenomas [34]. Therefore, we speculated that angiogenesis is associated with the morphogenesis of OVX via an unknown mechanism. Additional molecular evidence is required to determine the manner in which angiogenesis affects the morphogenesis of OVX.

The difficulty in establishing a strict distinction between M1 and M2 macrophages was another issue that was carefully considered in our study. It has been reported that no suitable immunohistochemical markers exist to detect M1 macrophages [30]. Moreover, CD68/STAT1 and CD163/STAT1 have been reported as M1 markers [35]. Therefore, the foamy macrophages of OVXs cannot be definitely used to classify M2 macrophages, even if they express CD163. On the other hand, studies have reported a few rare cases of OVX associated with malignant lesions [36,37,38]. In addition, several investigators have reported about the correlation between CD163^+^ macrophages and epithelial dysplasia and the malignancy of oral precancerous lesions [13,14,39,40]. Therefore, we must not overlook the malignant potential of squamous epithelium within the OVXs, which are considered as benign reactive lesions. It may be necessary to undertake a prospective study in order to clarify the malignant transformation of OVX.

## 5. Conclusions

To the best of our knowledge, this is the first report to demonstrate that the foamy macrophages expressing CD163 are associated with the morphogenesis of OVX through the process of angiogenesis by VEGF expression.

## Figures and Tables

**Table 1:** Clinical and histological characteristics of patients with oral verruciform xanthoma.

Case	Sex	Age (Years)	Region	Size (mm)	Clinical Diagnosis	Histological Classification	Previous Medical History
1	M	15	Gingiva	5.0	Epulis	Verrucous	Pneumonia Macroscopic hematuria
2	M	54	Gingiva	5.0	Gingival tumor	Flat	Dyslipidemia Hyperuricemia
3	M	43	Palate	3.0	Papilloma	Flat	None
4	F	48	Gingiva	4.0	Erosion	Flat	Appendicitis Basedow’s disease
